# RETRACTED: Rauf et al. Neuroinflammatory Markers: Key Indicators in the Pathology of Neurodegenerative Diseases. *Molecules* 2022, *27*, 3194

**DOI:** 10.3390/molecules31060951

**Published:** 2026-03-12

**Authors:** Abdur Rauf, Himani Badoni, Tareq Abu-Izneid, Ahmed Olatunde, Md. Mominur Rahman, Sakshi Painuli, Prabhakar Semwal, Polrat Wilairatana, Mohammad S. Mubarak

**Affiliations:** 1 Department of Chemistry, University of Swabi, Anbar 23561, Khyber Pakhtunkhwa, Pakistan; 2 Department of Biotechnology, School of Applied and Life Sciences, Uttaranchal University, Premnagar, Dehradun 248006, India; himani318@gmail.com; 3 Pharmaceutical Sciences Department, College of Pharmacy, Al Ain University for Science and Technology, Al Ain 64141, United Arab Emirates; tizneid@gmail.com; 4 Department of Medical Biochemistry, Abubakar Tafawa Balewa University, Bauchi 740272, Nigeria; olatundebch@gmail.com; 5 Department of Pharmacy, Faculty of Allied Health Sciences, Daffodil International University, Dhaka 1207, Bangladesh; mominur.ph@gmail.com; 6 Uttarakhand Council for Biotechnology (UCB), Premnagar, Dehradun 248007, India; sakshipainulii@gmail.com; 7 Department of Life Sciences, Graphic Era (Deemed To Be University), Dehradun 248002, India; semwal.prabhakar@gmail.com; 8 Department of Clinical Tropical Medicine, Faculty of Tropical Medicine, Mahidol University, Bangkok 10400, Thailand; 9 Department of Chemistry, The University of Jordan, Amman 11942, Jordan

The journal retracts the review article titled “Neuroinflammatory Markers: Key Indicators in the Pathology of Neurodegenerative Diseases” [1], cited above.

Following publication, concerns were brought to the attention of the publisher regarding an overlap between this publication and previously published review articles, produced by different authorship groups.

In accordance with the journal’s standard procedures, an investigation was conducted by the Editorial Office, significant overlap was identified between this publication [1] and two previously published review articles [2,3], without appropriate acknowledgement or citation. Consequently, the Editorial Board has decided to retract this publication, as per MDPI’s retraction policy.

This retraction was approved by the Editor-in-Chief of the journal *Molecules*.

The authors have been informed of this retraction and have indicated their disagreement with this decision.
